# Histopathological Evaluation of Endometrial Patterns in Women With Postmenopausal Bleeding and Their Correlation With Clinical and Radiological Features

**DOI:** 10.7759/cureus.106028

**Published:** 2026-03-28

**Authors:** Pooja Yadav, Loveleen Kaur, Permeet K Bagga, Suparna Grover, Mandeep Randhawa, Jaspreet Singh

**Affiliations:** 1 Department of Pathology, Government Medical College and Guru Nanak Dev Hospital, Amritsar, IND; 2 Department of Obstetrics and Gynaecology, Government Medical College and Guru Nanak Dev Hospital, Amritsar, IND

**Keywords:** endometrial hyperplasia, endometrial thickness, gynaecology, histopathology, postmenopausal bleeding

## Abstract

Background

Postmenopausal bleeding (PMB) is a common gynaecological complaint and an important clinical indicator of underlying endometrial pathology, including malignancy. The present study aimed to evaluate histopathological patterns in women presenting with PMB and to correlate these findings with clinical and radiological parameters, particularly endometrial thickness (ET).

Materials and methods

This one-year observational study included 35 postmenopausal women presenting with bleeding per vaginum. Endometrial tissue obtained via dilation and curettage, pipelle biopsy, or hysterectomy was subjected to histopathological evaluation. Clinical details and transvaginal ultrasonographic ET measurements were recorded. Statistical analysis was performed using a chi-square (χ²) or Fisher’s exact test, with a p-value of < 0.05 considered significant.

Results

The mean age of the patients was 54.77 ± 6.4 years. Most women were aged 50-59 years (40%), multiparous with parity of two (45.7%), and from urban areas (80%). Histopathological examination revealed benign lesions in 26 cases (74.3%), premalignant lesions in four cases (11.4%), and malignant lesions in five cases (14.3%). Atrophic endometrium was the most common finding (28.6%), followed by endometrial hyperplasia without atypia (20%). ET ranged from <4 mm to >11 mm, with 40% of cases demonstrating ET ≤4 mm. Notably, no premalignant or malignant lesion was identified in cases with ET <4 mm. A statistically significant association was observed between increasing ET and premalignant/malignant histopathology (χ² = 8.90, p = 0.01).

Conclusion

In this cohort of women with PMB, benign endometrial pathology predominated (74.3%), with atrophic endometrium being the most frequent diagnosis (28.6%). Premalignant or malignant pathologies, in total, accounted for 25.7% of the women with PMB. Increasing ET was significantly associated with these lesions, whereas no such cases were observed with ET ≤4 mm. These findings support the role of ET as a stratification parameter in guiding histopathological evaluation of PMB.

## Introduction

Postmenopausal bleeding (PMB) refers to any instance of vaginal bleeding taking place 12 months or more after a woman's last menstrual period [1]. PMB can range anywhere from infrequent spotting per vaginum to scanty, moderate, or profuse bleeding per vaginum spanning over a variable number of days or weeks. It has a worldwide prevalence, accounting for approximately 10% of all gynaecological ailments [2]. After menopause, neither hormone-induced physiological bleeding nor dysfunctional/hormone-independent or abnormal bleeding per vaginum is expected to occur [3].

Several risk factors have been implicated in the occurrence of PMB, including the unregulated use of estrogen in hormone replacement therapy (HRT), nulliparity status, late onset of menopause, and the combination of systemic conditions such as obesity, hypertension, and diabetes [4,5]. All of these are also recognised as risk factors for the development of endometrial malignancies. However, the evaluation of these risk factors requires detailed clinical and anthropometric data, which may not always be available in resource-limited settings. The blend of these risk factors can lead to pathologies including uterine adenomyosis, cervical polyps, cervical cancer, vaginal cancer, and tubal and ovarian cancer. Other than these afflictions, some pathologies amounting to clinical PMB include age-related endometrial atrophy, inflammatory endometritis, proliferative conditions such as endometrial hyperplasia or endometrial polyps, and uterine cancer [4].

Though the likelihood of PMB decreases with age, the risk of associated malignancy increases as age advances, and the time between menopause and the onset of PMB lengthens [6]. The current diagnostic evaluation includes clinical examination, cervical smear testing, pelvic ultrasonography (for endometrial thickness (ET)), diagnostic hysteroscopy, and endometrial tissue sampling. Of these, endometrial tissue sampling, either via biopsy, dilation and curettage (D&C), or hysteroscopy, is considered the gold standard for definite diagnosis as it is confirmatory in differentiating between normal and abnormal endometrial tissue [7].

While the association between increased ET and malignant pathology is widely recognised, the optimal ET threshold for predicting premalignant and malignant lesions remains debated, particularly across different populations [8]. Additionally, in resource-limited settings, where access to advanced diagnostic modalities may be constrained, identifying reliable clinical and radiological correlates that can aid in risk stratification becomes especially important. However, data evaluating the relationship between ET, demographic characteristics, and histopathological outcomes in such settings remain insufficient [9].

Through this study, we aimed to evaluate the spectrum of histomorphological endometrial patterns in women presenting with PMB at a tertiary care centre in North India and to analyse their correlation with clinical characteristics and ET. By quantifying the proportion of benign, premalignant, and malignant lesions and examining their association with radiological findings, this study aimed to contribute region-specific evidence that may assist in stratifying risk and guiding diagnostic evaluation in women with PMB.

## Materials and methods

This single-centre, hospital-based, cross-sectional observational study was conducted over a period of one year (August 2023 to July 2024) in the Department of Pathology at Government Medical College, Amritsar, Punjab, India, after obtaining institutional ethical clearance (approval number: BFUHS/2K25p-TH/1884). The study included endometrial sampling from all consecutive postmenopausal females presenting with PMB during the period. Postmenopausal status for the study cases was defined as any amount of bleeding per vaginum that occurred at least 12 months after their last menstrual period.

Women undergoing HRT or anticoagulant therapy or those with a known bleeding or coagulation disorder, prior gynaecological surgery, premature ovarian failure, radiation or chemotherapy resulting in menopause, or those already diagnosed as cases of cervical, vaginal, vulval, or uterine malignancy were excluded from the study. The total sample size of 35 cases was determined by the number of eligible cases presenting during the study period and by including all the consecutive patients meeting the inclusion criteria.

All the study cases were explained in their vernacular language about the procedure to be followed, and their written informed consent was taken. A detailed clinical history was recorded, including patient age, residence (urban or rural), age at menopause, duration since menopause, parity, presenting bleeding pattern (spotting, scanty, moderate, or profuse bleeding), and relevant medical history such as diabetes mellitus, hypertension, any medication use, and family history of gynaecological malignancies. ET was measured via transvaginal ultrasonography and recorded in millimetres (mm). For all the cases, a standardised maximum ET in the sagittal plane was recorded. This was followed by endometrial tissue sampling, obtained at the department of gynaecology through modes of D&C, pipelle biopsy, or hysterectomy, and later submitted for histopathology evaluation at the department of pathology.

Histopathological assessment was performed independently by two pathologists on 3-5 micrometre-thick representative tissue sections after subsequent staining with haematoxylin and eosin (H&E) stain. The findings were compared. In cases of discordant findings, the slides were jointly reviewed to reach a consensus after discussion. The categorisation of the histopathological diagnosis reached was done according to the criteria outlined in the World Health Organisation (WHO) Classification of Female Genital Tumours, 2020 [10]. Based on these criteria, the cases were categorised into benign, premalignant (endometrial hyperplasia with or without atypia), or malignant (endometrial carcinoma and its histological subtypes).

For analysis, the patient’s demographic, clinical, radiological and histopathological findings were compiled and organised into tables, followed by descriptive statistics to calculate frequencies, percentages, and mean ± standard deviation (SD) for continuous variables. Categorical variables were analysed using the chi-square test (χ²) or Fisher’s exact test where appropriate, using IBM SPSS Statistics software version 20 (IBM Corp., Armonk, NY, USA). A p-value < 0.05 was considered significant.

## Results

Demographic and clinical patient profile

A total of 35 female patients presenting with PMB and whose endometrial tissue sample was adequate for histopathological examination were included in the study. The mean age of the study cases was observed to be 54.77±6.4 years, with ages ranging from 40 years to 65 years. The largest proportion of cases belonged to the 50-59 years age group (n = 14; 40%), followed by ≥60 years (n = 13; 37.0%) and 40-49 years (n = 8; 22.8%). 

With regard to residence, 28 patients (80%) were from urban areas, while seven patients (20%) belonged to rural areas.

On probing the patient's past, obstetric, and personal histories, the mean age of menopause was noted as 46.83±3.5 years. The duration between menopause and the onset of PMB was ≥10 years in 14 cases (40%), one to three years in 12 cases (34.3%), four to six years in six cases (17.1%), and seven to nine years in three cases (8.6%). Regarding obstetric history, the highest proportion of cases had parity of two (n = 16; 45.7%), followed by nulliparous women (n = 8; 22.8%), parity of three (n = 6; 17.1%), and parity greater than three (n = 5; 14.3%).

Comorbid conditions were present in eight patients (22.9%), including diabetes mellitus, hypertension, or both, while 27 patients (77.1%) had no reported systemic comorbidities. A family history of genitourinary malignancy was reported in one case (2.9%). Due to the small number of cases and lack of detailed stratification, no further statistical analysis was performed to assess their association with histopathological outcomes. Additionally, body mass index (BMI) was not recorded in this study, precluding evaluation of comorbid conditions or obesity as a risk factor.

Endometrial thickness

ET measured by transvaginal ultrasonography ranged from <4 mm to >11 mm. The largest proportion of cases demonstrated ET between 4-11 mm (n = 17; 48.6%), followed by ET ≤4 mm (n = 14; 40%), and ET >11 mm (n = 4; 11.4%).

Histopathological features

Histopathological examination revealed eight distinct endometrial patterns. The distribution of histomorphological diagnoses is presented in Table 1.

**Table 1:** Histomorphological diagnosis in the study

Histomorphological diagnosis	Cases (n)	Percentage (%)
Atrophic endometrium	10	28.57
Disordered proliferative phase endometrium	5	14.29
Chronic endometritis	1	2.86
Pseudodecidual stromal change	1	2.86
Endometrial hyperplasia without atypia	7	20.0
Endometrial hyperplasia with chronic endometritis	2	5.7
Endometrial hyperplasia with atypia	4	11.43
Endometrial carcinoma (adenocarcinoma, endometrioid carcinoma)	5	14.29
Total cases	35	100.00

Overall, 26 cases (74.3%) were categorised as benign, four cases (11.4%) as premalignant, and five cases (14.3%) as malignant lesions.

Among the benign lesions, atrophic endometrium was the most frequent diagnosis (n = 10; 28.6%), followed by endometrial hyperplasia without atypia (n = 7; 20%) and disordered proliferative phase endometrium (n = 5; 14.3%). Less frequent findings included chronic endometritis and pseudodecidual stromal change.

Among premalignant lesions, endometrial hyperplasia with atypia was identified in four cases (11.4%). Malignant lesions included endometrial carcinoma (endometrioid adenocarcinoma) in five cases (14.3%).

**Figure 1:**
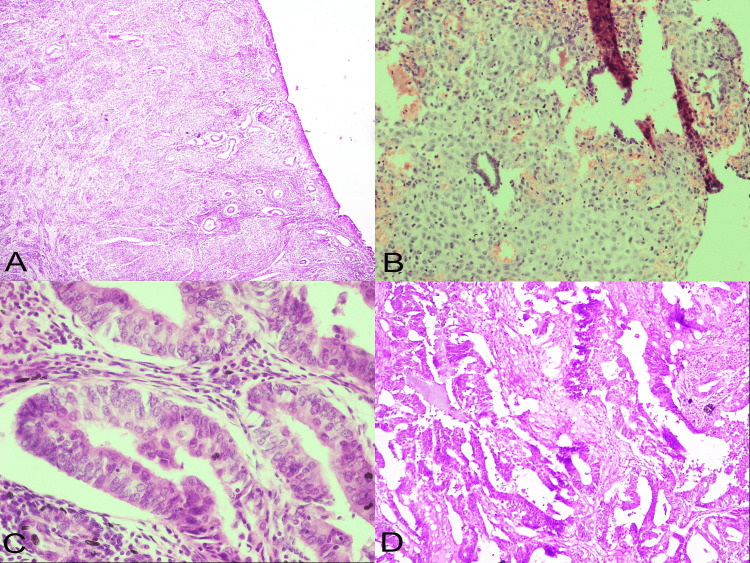
Microphotographs of endometrial histomorphological patterns A. Atrophic endometrium, widely spaced small, inactive endometrial glands lined by low columnar epithelium embedded in fibrotic endometrial stroma (H&E, x40); B. Pseudodecidualised stromal change, occasional inactive endometrial glands embedded within pseudodecidualised endometrial stroma with abundant cytoplasm (H&E, x400); C. Endometrial hyperplasia with atypia, closely arranged endometrial glands with decreased intervening stroma. The glands are displaying tufting, loss of nuclear polarity and rounded nuclei with opened-up chromatin and conspicuous nucleoli (H&E, x400); and D. Endometrial carcinoma, closely back-to-back and arborising endometrial glands lined by atypical cells with scant to absent intervening stroma (H&E, x100). H&E: haematoxylin and eosin

Representative microphotographs of selected histopathological patterns are illustrated in Figure 1.

Findings from statistical analysis

The relationship between ET and histopathological diagnosis demonstrated a statistically significant association. No premalignant or malignant lesions were identified in patients with ET ≤4 mm (n = 14). Among patients with ET between 4-11 mm, eight of 17 cases (47.1%) demonstrated premalignant or malignant pathology. Among patients with ET >11 mm, one of four cases (25%) showed malignant pathology. Statistical analysis demonstrated a significant association between increasing endometrial thickness and premalignant/malignant lesions (χ² = 8.90; p = 0.01).

In contrast, other evaluated variables-including age group, parity, and duration since menopause-did not demonstrate a statistically significant association with histopathological outcome (p-value = 0.99, 0.75, and 0.73, respectively).

Table 2 summarises the association between clinical and radiological characteristics and the histopathological diagnosis (benign versus premalignant/malignant).

**Table 2:** Association of various clinical and radiological characteristics with benign and premalignant/malignant histological diagnosis in the study P-values are based on the chi-square (χ²)​ test; ​a ​​​​ p-value < 0.05 was considered significant. df: degree of freedom

Characteristic	Benign (n=26)	Premalignant/malignant (n=9)	Chi-square value (χ²); Degree of freedom (df)	P-value
Age groups (years)			χ² = 0.43; df = 2	0.99
40-49	6	2		
50-59	11	3		
≥ 60	9	4		
Parity			χ² = 1.02; df = 4	0.75
0	7	1		
1	0	0		
2	11	5		
3	4	2		
>3	4	1		
Time since menopause (years)			χ² = 1.17; df = 3	0.73
1-3	9	3		
4-6	4	2		
7-9	3	0		
≥ 10	10	4		
Endometrial thickness (millimetres)			χ² = 8.90; df = 2	0.01
<4 mm	14	0		
4-11	9	8		
>11 mm	3	1		

## Discussion

Gynaecological complaints, especially PMB, have accelerated among females after menopause, with malignancy being the most dreaded underlying pathology. However, the higher cure rates and better overall survival of females associated with early detection and treatment of endometrial carcinoma emphasise the need for increased clinician awareness and pathological correlation in such patients [11,12].

The present study included 35 consecutive postmenopausal female patients presenting with PMB, where identification of the various endometrial histomorphological patterns and their segregation into benign, premalignant, and malignant pathologies was performed. The majority of women (40% of the total) presented with PMB between 50 and 59 years of age, with the mean age of presentation as 54.77±6.4 years. This was concordant with reported literature [4,13,14]. This observation reflects the increasing frequency of clinical presentation during the early postmenopausal years. 

Predominant cases in this study hailed from urban areas, similar to findings by studies conducted by Kothapally et al. and Dasgupta et al. [15,16]. These findings could likely be owing to the better availability of health services in the urban locations.

Most of the women in this study were multiparous, with parity of two being the most frequent obstetric profile. Although previous studies have suggested an inverse association between parity and the risk of endometrial pathology [17,18], the present study did not demonstrate a statistically significant relationship between parity and histopathological diagnosis (p = 0.75). The limited sample size and absence of patients with parity of one in the present study may have influenced this finding. 

The time duration between the onset of menopause and PMB in a major proportion of cases (14 cases of the total) amounted to a decade or more. No statistically significant association was observed between duration since menopause and histopathological outcome (p = 0.73). The published literature reports a fluid trend regarding the time since menopause and incidence of PMB [19,20].

In this study, about three of five cases had no identifiable systemic risk factors such as diabetes, hypertension, or both. These results align with the study by Begum and Samal [14], who reported similar patterns. Although these systemic risk factors are implicated in endometrial pathologies, their role could not be adequately assessed in the present study, owing to their incidence only in limited cases and a non-stratified documentation manner, precluding meaningful statistical analysis. Additionally, body mass index, a key parameter for evaluating obesity, was not recorded. Therefore, no definitive conclusions can be drawn regarding their association with histopathological outcomes in this cohort. Future studies incorporating detailed metabolic and anthropometric profiling are required to better elucidate these relationships. Familial history revealed only an isolated case with the incidence of genitourinary cancer. However, these findings did not prove to be significant.

ET assessment proved to be a notable parameter in implicating the histological diagnosis, as well as differentiating between hyperplastic and malignant cases. In our study, no instance of premalignant or malignant endometrial pathology was observed when ET was below 4 mm. This finding is concordant with the reported literature [21].

Histopathological examination remains the cornerstone for identifying the underlying aetiology of PMB. A total of eight distinct entities were observed in this study, segregated into three categories: benign lesions constituting the majority of cases (74.3%), followed by premalignant and malignant lesions accounting for 11.4% and 14.3% of cases, respectively. Among benign lesions, atrophic endometrium was the most common histopathological finding (28.6%), followed by endometrial hyperplasia without atypia (20%) and disordered proliferative endometrium (14.3%). These findings are in keeping with the results reported by Gredmark et al. and Kumari et al. [22,18] that reported atrophic endometrium as the most common cause of PMB, while another study by Talwar et al. [13] found hyperplasia without atypia to be the most frequent in PMB cases.

The premalignant and malignant categories, respectively accounting for 11.4% and 14.3% of the total study cases, highlight the clinical significance of evaluating postmenopausal bleeding, as a considerable proportion of patients may harbour premalignant or malignant pathology.

The assessment of ET by transvaginal ultrasonography demonstrated a significant association with histopathological outcome (p = 0.01). Notably, no premalignant or malignant lesions were identified in patients with endometrial thickness ≤4 mm, whereas a higher proportion of such lesions was observed among patients with greater endometrial thickness. These findings support the utility of ET as a useful screening and risk-stratification parameter in PMB cases. Similar observations have been reported in previous studies, which have suggested that a threshold of 4 mm or less is associated with a low risk of endometrial malignancy [20,23].

Despite these observations, it is important to emphasise that histopathological evaluation remains the definitive gold standard diagnostic modality for establishing the exact aetiology of PMB. Radiological assessment alone cannot reliably distinguish between different endometrial pathologies, particularly premalignant lesions.

The present study has several strengths. First, it utilises histopathological evaluation, the gold standard for diagnosing endometrial pathology, thereby ensuring diagnostic accuracy. Second, the study incorporates a combined clinicoradiological-pathological approach, allowing meaningful correlation between ET and histological findings. Third, the use of standardised diagnostic criteria based on the WHO 2020 classification enhances reproducibility and comparability with other studies [10]. Additionally, the study contributes region-specific data from a tertiary care centre in North India, where such data remain relatively limited.

However, the study also has certain limitations that need to be acknowledged. The small sample size, single-centre design and inadvertent selection bias, along with the lack of follow-up data, may underpower the study for multivariable analysis and the generalisability of the findings. The predominance of urban patients in this cohort further limits the applicability of the findings to rural populations. The absence of body mass index data and the limited assessment of metabolic comorbidities (diabetes and hypertension) restricted the evaluation of established risk factors. The pathologists evaluating the histological sections were not blinded to the case's clinical/radiological details (age, postmenopausal status, parity and ET), leading to a potential observer bias. Finally, although ET was shown to correlate with histopathological findings, transvaginal ultrasonography is operator-dependent and may be subject to interobserver variability, which was not assessed in this study. Larger multicentre studies with a greater number of patients may provide more robust data regarding the clinicopathological spectrum of PMB.

## Conclusions

In this cohort of women presenting with PMB, benign endometrial pathology predominated, with atrophic endometrium being the most frequent histopathological finding. ET measured by transvaginal ultrasonography emerged to play a vital role as an initial, non-invasive and reliable screening tool to identify women at low risk for significant endometrial pathology, thereby aiding clinical decision-making and reducing the need for unnecessary invasive procedures.

Although a lower ET may indicate a reduced likelihood of significant pathology, histopathological evaluation remains the definitive diagnostic method for determining the underlying cause of postmenopausal bleeding. The findings of this study should be interpreted in light of its limitations, including the small sample size and low count of malignant cases, and single-centre design, which may limit generalizability and necessitate caution while interpreting the observations. Larger multicenter studies are required to further clarify the clinicopathological associations in women presenting with PMB.
